# Parental Supervision and Its Relation With Emotional and Behavioral Problems in Secondary School Children

**DOI:** 10.7759/cureus.35291

**Published:** 2023-02-22

**Authors:** Nikhita Das, Sabita Dihingia, Dhrubajyoti Bhuyan, Kavery Bora

**Affiliations:** 1 Department of Psychiatry, Assam Medical College and Hospital, Dibrugarh, IND; 2 Department of Psychiatry, Nalbari Medical College and Hospital, Nalbari, IND

**Keywords:** positive parenting, child monitoring, child and adolescent, delinquency, parenting style

## Abstract

Background

Emotional and behavioral problems of children are a common concern for parents and mental health stakeholders alike. Poor parenting is a well-known factor associated with behavioral problems in children. There is unanimity regarding the correlation between parental supervision and emotional and behavioral problems. This present study aimed to establish a relationship between parental supervision and emotional and behavioral problems, as it could make way for further research based on the concept of parental supervision, which parents can quickly adopt as an intervention strategy for children with behavioral and emotional problems.

Aim

We aim to assess parental supervision and its relation with emotional and behavioral problems in secondary school children.

Method

This is a community-based cross-sectional observational study among 770 parents of children from schools in Dibrugarh, Assam, over a period of one year. Multistage random sampling was applied to obtain the sample size. The Strengths and Difficulties Questionnaire (SDQ) was used to assess children’s emotional and behavioral problems, the Alabama Parenting Questionnaire (APQ) was used to assess parental supervision, and sociodemographic proforma was used to study various demographic variables. The observed data were analyzed using the computer program Statistical Package for the Social Sciences for Macintosh version 24.0 (IBM SPSS Statistics, Armonk, NY, USA).

Results

The study revealed that participants’ poor supervision had a positive correlation with emotional and behavioral problems. Poor monitoring/supervision had a positive correlation with total difficulty score levels, and positive parenting practices such as involvement and positive parenting had a negative correlation with emotional and behavioral problems. There was a statistically significant association between behavioral problems and selected demographic variables such as parents’ education, socioeconomic status, and family type. The study also found that there was a significant statistical association between sociodemographic variables such as age and negative parenting practices such as poor monitoring/supervision, inconsistent discipline, and corporal punishment.

Conclusion

It was found that factors such as inconsistent discipline and poor supervision had a significant impact on emotional and behavioral problems in children. In future monitoring research, one can adopt a constructional approach, where the goal should be to explain and distinguish good parental supervision behaviors from poor supervision. This knowledge can be used to develop good intervention strategies to halt such emotional and behavioral problems.

## Introduction

A child undergoes various physical, emotional, and social stages from birth to adulthood. Emotional and behavioral problems in a child develop from any interference in their mental development. Most issues commence during childhood and have significance for later life, such as scholastic abilities, learning new skills, using a substance, engaging in violent activities, and developing warm relations, and tend to continue into adulthood [1]. These emotional and behavioral problems of children are a common concern for parents and mental health professionals. These problems form a significant portion of psychiatric disorders in children.

According to Malhotra (1992), the percentage of children living in developing countries is around 80%, with poor mental healthcare services [2]. A review of Indian studies reported that the prevalence of mental health problems in school-going children ranges from 6.33% to 43.1% [1]. Unfortunately, the majority of parents, teachers, relatives, and other adults fail to perceive such subtle behavioral changes. Early improvement is encouraged by early identification, and a healthier and adaptive path is achieved by pushing such evolving course. Regarding supervision and parenting practices, studies have highlighted the significance of parental supervision in the progress and independence of the child. However, it remains a debatable component owing to its complex nature. There is unanimity regarding the negative correlation between parental supervision and emotional and behavioral problems [3].

The study focuses on the relationship between parental supervision and emotional and behavioral problems in school children in the northeastern part of India, which has not been extensively analyzed before. Additionally, the study proposes parental supervision as a potential intervention strategy for children with behavioral and emotional problems, which is a unique approach that could pave the way for further research and interventions.

## Materials and methods

**Figure 1:**
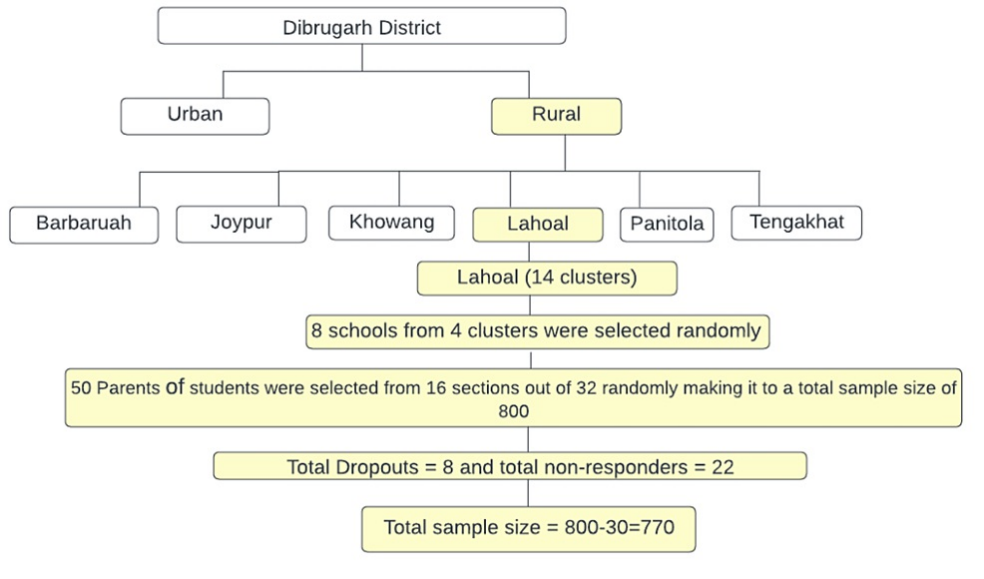
Flowchart showing multistage sampling

This is a community-based cross-sectional study carried out in eight schools in Dibrugarh over a period of one year. A multistage random sampling technique was applied to obtain a 770 sample size. The study participants were selected using multistage random sampling as shown in Table 1 and Figure 1.

**Table 1:** Stages of multistage sampling

Stage	Description
Stage 1	There are six rural blocks in Dibrugarh district, Lahoal, Panitola, Khowang, Barbaruah, Joypur, and Tengakhat, out of which one block (Lahoal) was randomly selected.
Stage 2	According to information provided by the inspector of schools, there are 14 clusters in Lahoal block, and four clusters, Tinkunia, Paltanbazar, Shankardev, and Maijan Niz kana, were selected randomly by lottery method.
Stage 3	In these selected clusters, a list of schools was prepared, from which two schools were selected randomly from each cluster, making a total of eight schools. From a total number of 32 sections, 16 sections were included.
Stage 4	Fifty parents of students were selected from every 16 sections according to inclusion criteria, eight sections each belonging to Class IX and Class X (8*2=16 sections, 50 parents*16 sections=800 parents).​​​​ With 22 nonresponders and eight dropouts, a sample size of 770 was obtained.

Inclusion criteria

Parents of students from Class IX and Class X of government and private schools from the rural area of Dibrugarh who gave their informed consent were included in this study.

Exclusion criteria

Parents and children suffering from any chronic debilitating mental/physical illness were excluded.

Tools

The tools used in the study comprised an informed consent form, sociodemographic proforma, Modified Kuppuswamy Socioeconomic Scale, Strengths and Difficulties Questionnaire (SDQ), and Alabama Parenting Questionnaire (APQ) (Appendices).

Sociodemographic Proforma

A self-designed proforma to collect sociodemographic details of the subject has been used. It was developed in the Department of Psychiatry, Assam Medical College and Hospital.

Modified Kuppuswamy Scale (2020) for Socioeconomic Status

This scale was used to find out to which class of socioeconomic status the parents and children belonged [4].

Strengths and Difficulties Questionnaire

Strengths and Difficulties Questionnaire is developed to assess behavioral problems in children and adolescents. It is a screening tool that is used among children and adolescents aged about two to 17 years old to assess their mental health. It exists in several versions that have been applied to various studies. All versions include 25 items on emotional and behavioral features [5].

Alabama Parenting Questionnaire

The Alabama Parenting Questionnaire (APQ) is a 42-item self-reporting assessment tool developed by Dr. Paul Frick. It has five theoretical constructs: parental involvement, positive parenting, poor monitoring/supervision, inconsistent discipline, and corporal punishment [6].

Procedure

After taking permission from the Office of Inspector of Schools, Dibrugarh, and the principal of all eight schools, brief interactions with the class teachers were held. Thereafter, the students were instructed about the study, and a manual was handed to them for their parents containing information about the study, anonymity, and implications. Written informed consent was obtained from parents after a week. The Modified Kuppuswamy Socioeconomic Scale was applied for assessing the socioeconomic class of parents. Permission for translation and use of the questionnaires was obtained from the authors. The questionnaires were translated to the target language, Assamese, and then, back translation was done by a different bilingual expert who was blinded to the source language. The questionnaires were validated. The Strengths and Difficulties Questionnaire was used to study emotional and behavioral problems, and the Alabama Parenting Questionnaire was used to assess parental supervision. The questionnaires were handed to the students with an instruction manual for the parents and collected after a period of seven days. The questionnaires were distributed to 800 students to obtain a sample of 770, considering dropouts and nonresponders. In this study, there were eight dropouts, and 22 nonresponses and incomplete responses.

Data analysis

The observed data were analyzed using the computer program Statistical Package for the Social Sciences for Macintosh version 24.0 (IBM SPSS Statistics, Armonk, NY, USA). Results were presented as proportions, ratios, percentages, and mean. The different statistical tests used in the study were unpaired t-test, chi-square test, and Pearson correlation coefficient. For all analyses, the statistical significance was fixed at a 5% level (p<0.05). The study was approved by the Institutional Ethics Committee of Assam Medical College bearing certificate number AMC/EC/PG/5612.

## Results

Table 2 shows that among 770 participants, the majority of fathers were in the age group of 41-50 years (50%) and the majority of mothers were in the age group of 31-40 years (67%). The majority of children were male (55%) and were in the age group of 15-16 years (51%). The study showed that most of the participants belonged to the upper-lower class of socioeconomic status (57%) and nuclear families (85%) and followed Hinduism (75%).

**Table 2:** Sociodemographic variables of the study participants (parents) and their children

Sociodemographic characteristics		Number	Percentage (%)
Age of father (in years)	<30	0	0
	31-40	311	40
	41-50	386	50
	51-60	60	8
	>60	13	2
Age of mother (in years)	<30	0	0
	31-40	519	67
	41-50	228	30
	51-60	23	3
	>60	0	0
Age of child (in years)	13-14	375	49
	15-16	395	51
Sex of child	Male	500	55
	Female	270	45
Type of family	Nuclear	654	85
	Joint	116	15
Socioeconomic class	Class 1	12	1.5
	Class 2	132	17
	Class 3	173	22.5
	Class 4	440	57
	Class 5	13	2
Religion	Hinduism	578	75
	Islam	174	43
	Christianity	11	1.4
	Other	8	0.6

The means±standard deviations for the Alabama Parenting Questionnaire in the subscales are as follows: 29.28±3.41 with a range of 19-58 for involvement, 19.51±2.84 with a range of 11-55 for positive parenting, 29.43±4.01 with a range of 18-40 for poor monitoring/supervision, 18.50±2.59 with a range of 10-36 for inconsistent discipline, and 3.95±1.54 with a range of 3-9 for corporal punishment (Table 3).

**Table 3:** Distribution showing Alabama Parenting Questionnaire score across secondary school children APQ: Alabama Parenting Questionnaire

Variables of APQ	Mean	Standard deviation	Maximum	Minimum
Involvement	29.28	3.41	58	19
Positive parenting	19.51	2.84	55	11
Poor monitoring/supervision	29.43	4.01	40	18
Inconsistent discipline	18.50	2.59	36	10
Corporal punishment	3.95	1.54	9	3

Table 4 shows that the mean±standard deviation for the variables of the Strengths and Difficulties Questionnaire of total difficulty score level by parents was 6.06±4.13, with the minimum score being 0 and the maximum being 20. The mean±standard deviation for the individual subscales for parents for emotional symptoms was 1.26±1.08, for conduct problems was 5.29±1.34, for hyperactivity was 1.77±1.64, and for peer problems was 3.71±2.81.

**Table 4:** Distribution showing Strengths and Difficulties Questionnaire score across secondary school children SDQ: Strengths and Difficulties Questionnaire

Variables of SDQ	Mean	Standard deviation	Maximum	Minimum
Emotional symptoms	1.26	1.08	5	0
Conduct problems	5.29	1.34	17	0
Hyperactivity	1.77	1.64	9	0
Peer problems	3.71	2.81	16	0
Total difficulty score	6.06	4.13	20	0

Table 5 shows that poor monitoring/supervision had a positive correlation with emotional symptoms (r=0.079, p=0.027), hyperactivity (r=0.134, p=0.001), peer problems (r=0.108, p=0.002), and total difficulty score (r=0.143, p=0.001), which were statistically significant. Positive parenting practices such as positive parenting had a negative correlation with conduct problems (r=-0.091, p=0.01) and total difficulty score (r=-0.062, p=0.041). Inconsistent discipline had a weak positive correlation with hyperactivity (r=0.058, p=0.01). Corporal punishment had no significant correlation with the total difficulty score.

**Table 5:** Correlation between different domains of positive and negative practices and emotional and behavioral problems (n=770) p: p value, p<0.05 (*significant at 0.05 level of significance), r: Pearson correlation coefficient

	Emotional symptoms		Conduct problems		Hyperactivity		Peer problems		Total difficulty score	
	r	p	r	p	r	p	r	p	r	p
Parental involvement	-0.059	0.098	0.004	0.899	-0.018	0.614	-0.027	0.445	-0.046	0.012
Positive parenting	-0.064	0.0738	-0.091	0.01*	-0.035	0.325	-0.033	0.359	-0.062	0.041*
Poor monitoring/supervision	0.079	0.027*	0.044	0.216	0.134	0.001*	0.108	0.002*	0.143	0.001*
Inconsistent discipline	-0.023	0.521	0.044	0.221	0.058	0.010*	0.059	0.075	0.033	0.346
Corporal punishment	0.019	0.581	-0.049	0.167	-0.028	0.430	-0.027	0.453	-0.012	0.719
Specific disciplinary practice	0.024	0.499	-0.033	0.360	0.021	0.550	0.024	0.502	0.029	0.418

Regarding emotional and behavioral problems, 14.06% of children had total difficulty score levels in the abnormal range (high and very high) with 14% at substantial risk for emotional symptoms, 43.5% for conduct problems, 34.1% for hyperactivity, and 15% for peer problems.

There was a significant statistical association between sociodemographic variables such as age and negative parenting practices such as poor monitoring/supervision (χ2=272, p<0.001), inconsistent discipline (χ2=305.73, p<0.001), and corporal punishment (χ2=83.32, p<0.001). Positive parenting practices such as positive parenting had a significant association with socioeconomic class (χ2=71.56, p<0.030). Education had an association with poor monitoring/supervision (χ2=275.72, p<0.001) and corporal punishment (χ2=45.09, p=0.038). The occupation of parents had an association with negative parenting practices such as inconsistent discipline (χ2=234.98a, p<0.001). Poor monitoring/supervision also had an association with income (χ2=198.78, p=0.012) and family type (χ2=53.82, p=0.001) (Table 6).

**Table 6:** Association of parenting practices with the participants’ sociodemographic variables p: p value, p<0.05 (*significant at 0.05 level of significance), χ2: Pearson’s chi-square

Sociodemographic variables	Parental involvement		Positive parenting		Poor monitoring/supervision		Inconsistent discipline		Corporal punishment	
	χ2	p	χ2	p	χ2	p	χ2	p	χ2	p
Age	98.62	105	117.01	0.012	274.03	<0.001*	305.73	<0.001*	83.32	<0.001*
Gender	27.37	0.159	11.32	0.839	23.56	0.371	27.47	0.051*	8.64	0.192
Religion	58.29	0.645	37.14	0.927	43.11	0.987	23.95	1.000	13.88	0.736
Education	85.02	0.924	71.09	0.860	275.72	<0.001*	67.67	0.916	45.09	0.038*
Occupation	194.9	0.076	150.89	0.181	189.54	0.230	234.98	<0.001*	48.61	0.448
Income	92.72	0.988	107.82	0.328	198.78	0.012*	151.32	<0.001	40.70	0.271
Socioeconomic class	66.44	0.359	71.56	0.030*	81.71	0.092	65.13	0.088	18.72	0.409
Family type	14.15	0.863	11.75	0.815	53.82	0.001*	22.18	0.178	9.49	0.147
Marital status	26.17	1.00	23.89	1.00	46.36	0.968	16.25	1.00	1.88	1.000

On studying the association between emotional and behavioral problems and sociodemographic variables, there was a statistically significant association between total difficulty score and selected demographic variables such as parents’ education (χ2=389.411, p=0.001), occupation (χ2=364.57, p=0.01), income (χ2=198.78, p=0.012), socioeconomic class (χ2=110.925, p=0.002), and family type (χ2=53.823, p=0.001). Parents’ ages had a statistically significant association with emotional symptoms (χ2=43.261). The subclass emotional symptoms also had an association with education (χ2=45.46, p=0.07), income (χ2=45.04, p=0.038), and family type of parents (χ2=29.27, p=0.01), which was found to be statistically significant (Table 7).

**Table 7:** Association of emotional and behavioral problems with participant’s sociodemographic variables p: p value, p<0.05 (*significant at 0.05 level of significance), χ2: Pearson’s chi-square

Sociodemographic variables	Emotional symptoms		Conduct problems		Hyperactivity		Peer problems		Total difficulty score	
	χ2	p	χ2	p	χ2	p	χ2	p	χ2	p
Age	84.89	0.001*	92.29	0.657	65.9	0.223	116.2	0.197	224.95	0.671
Gender	8.523	0.130	16.18	0.063	12.19	0.206	14.42	0.701	20.21	0.781
Religion	16.36	0.358	55.55	0.826	29.83	0.322	63.12	0.185	82.48	0.343
Education	45.46	0.007*	51.42	0.237	210.72	0.001*	278.61	0.001*	389.41	0.001*
Occupation	49.13	0.153	187.58	0.001*	373.46	0.001*	185.62	0.011*	364.57	0.001*
Income	45.04	0.038*	136.02	0.001*	381.47	0.001*	166.84	0.001*	198.78	0.012*
Socioeconomic class	24.92	0.051*	169.84	0.001*	21.33	0.770	59.34	0.287	110.92	0.002*
Family type	29.27	0.001*	6.73	0.664	28.969	0.001*	34.26	0.012*	53.82	0.001*
Marital status	0.069	0.874	20.02	0.830	17.498	0.918	65.57	0.134	75.95	0.545

## Discussion

The present study was a community-based cross-sectional study carried out in eight schools in Dibrugarh from July 2021 to June 2022 with a sample size of 770 to assess parental supervision and its relation with emotional and behavioral problems.

A prevalence of 14.04% of behavioral and emotional problems was found in the studied sample of children. When compared to the study done by Wang et al. in Beijing (1989) [7], who reported a prevalence rate of around 7%-8%, we found that the prevalence in this study was slightly higher. One possible reason could be the reported marginal increase observed in time trends due to factors such as changing family structure, increasing urbanity, and parenting styles. Another reason could be the selection of our sample where a school catering to predominantly lower socioeconomic status was chosen, and it is well known from the literature that children from lower and middle socioeconomic status tend to have more observed problems compared to children from higher socioeconomic status [8].

Another study conducted by Ahmed et al. (2021) reported the prevalence rate to be 77.5%, which was much higher compared to our study [9]. The possible reason can be that the participants were children who were chosen from a tertiary mental healthcare center and were already diagnosed with a mental disorder, whereas we have attempted to screen children in the community and those with no previous diagnosis of mental disorder. A study from Singapore reported a rate of behavioral and emotional problems in children of 12.5%, which was similar to that of our study [10].

The mean±standard deviation for the SDQ for the total difficulty level scored by parents was 6.06±4.13, with the minimum score being 0 and the maximum being 20. The mean±SD for the individual subscales for parents for emotional symptoms, conduct problems, hyperactivity, and peer problems was similar to the findings in the studies conducted by Bryant et al. (2020) [11] and Ortuño-Sierra et al. (2015) [12].

A positive correlation was found between poor supervision and emotional and behavioral problems. Poor monitoring/supervision (r=0.143, p=0.001) had a positive correlation with the total difficulty score, which was statistically significant. Positive parenting practices such as involvement (r=-0.046, p=0.01) and positive parenting (r=-0.062, p=0.05) had a negative correlation with emotional and behavioral problems. Corporal punishment had no significant correlation with the total difficulty score. This is in accordance with other studies where they have found that when the negative parenting score increases, the total difficulty score also increases, indicating that behavioral problems in children also increase [13,14]. Positive parenting practices had a negative correlation with emotional and behavioral problems, and this was in accordance with a study that found similar results [15].

In this study, we assessed associations using the chi-square test and found that positive parenting practice had an association with socioeconomic class (chi-square=71.56, p=0.03). In the case of negative parenting practices, we found that poor monitoring/supervision had a statistically significant association with age group (chi-square=274.03), education (chi-square=275.72), and family type (chi-square=53.82). Inconsistent discipline had a significant association with age (chi-square=305.73), gender (chi-square=24.47), and parental occupation (chi-square=234.98). This is in accordance with a study where they found an association between sociodemographic variables such as age and gender and negative parenting practices such as poor supervision and inconsistent discipline [16].

The study also found that emotional and behavioral problems had an association with sociodemographic variables. The total difficulty score level had an association with parents’ education, socioeconomic status, and family type, which was statistically significant. Conduct problems had an association with socioeconomic status (chi-square=169.84), and hyperactivity had an association with education (chi-square=210.72) and occupation of the parents (chi-square=372.46). This is in line with a study conducted by Feng et al., where they found that sociodemographic factors such as occupation and socioeconomic class had an association with emotional and behavioral problems in children [17].

The study however had its limitations. We have tried to assess if there was a relationship between parental supervision and emotional and behavioral problems in children. Whether a causal relationship exists or not was not studied. As self-reporting questionnaires were used in this study, there can be a probability that parents might have given more acceptable or socially desirable answers. The sample selection was based only on the rural blocks of Dibrugarh district and did not include urban blocks; therefore, the study results cannot be generalized.

## Conclusions

Our study revealed that poor parental supervision had a positive correlation with emotional and behavioral problems. Therefore, factors such as inconsistent discipline and poor monitoring had a significant impact on secondary school children. Parents who have practiced positive parenting had children with fewer or no behavioral problems. This finding could be useful in clinical practice where interventions based on parental supervision for emotional and behavioral problems in children can be attempted. In the future, one can adopt a constructional approach, where the goal should be to explain and distinguish good parental supervision behaviors from poor supervision. Education for parents may include supervision behaviors, for example, setting limits and restrictions, knowing about their whereabouts and the company they have, asking them about their activities, listening to their emotional turmoil, and encouraging disclosure. In this way, parental supervision may help in providing information to parents and professionals on appropriate ways to prevent such emotional and behavioral problems in children.

## Appendices

Table 8 and Table 9 show the Strengths and Difficulties Questionnaire and Alabama Parenting Questionnaire items, respectively.

**Table 8:** Strengths and Difficulties Questionnaire

Strengths and Difficulties Questionnaire		Not true	Somewhat true	Certainly true
1	Considerate of other people’s feeling			
2	Restless, overactive, cannot stay still for long			
3	Often complaints of headache, stomachaches, or sickness			
4	Shares readily with other young people, for example, CDs, games, and food			
5	Often loses temper			
6	Would rather be alone than with other young people			
7	Generally well-behaved, usually does what adults request			
8	Many worries or often seems worried			
9	Helpful if someone is hurt, upset, or feeling ill			
10	Constantly fidgeting or squirming			
11	Has at least one good friend			
12	Often fights with other young people or bullies them			
13	Often unhappy, depressed, or tearful			
14	Generally liked by other young people			
15	Easily distracted, concentration wanders			
16	Nervous in new situations, easily loses confidence			
17	Kind to younger children			
18	Often lies or cheats			
19	Picked on or bullied by other young people			
20	Often volunteers to help others			
21	Thinks thing out before acting			
22	Steals from home, school, or elsewhere			
23	Gets along better with adults than with young people			
24	Many fears, easily scared			
25	Good attention span, sees chores or homework through to the end			

**Table 9:** Alabama Parenting Questionnaire

Alabama Parenting Questionnaire		Never	Almost never	Sometimes	Often	Always
1	You have a friendly talk with your child					
2	You let your child know when he/she is doing a good job with something					
3	You threaten to punish your child and then do not actually punish him/her					
4	You volunteer to help with special activities that your child is involved in (such as sports and boy/girl scouts)					
5	You reward or give something extra to your child for obeying you or behaving well					
6	Your child fails to leave a note or let you know where he/she is going					
7	You play games or do other fun things with your child					
8	Your child talks you out of being punished after he/she has done something wrong					
9	You ask your child about his/her day					
10	Your child stays out in the evening past the time he/she is supposed to be home					
11	You help your child with his/her homework					
12	You feel that getting your child to obey you is more trouble than it’s worth					
13	You compliment your child when he/she does something well					
14	You ask your child what his/her plans are for the coming day					
15	You drive your child to a special activity					
16	You praise your child if he/she behaves well					
17	Your child is out with friends you don’t know					
18	You hug or kiss your child when he/she does something well					
19	Your child goes out without a set time to be home					
20	You talk to your child about his/her friends					
21	Your child is out after dark without an adult with him/her					
22	You let your child out of a punishment early					
23	Your child helps plan family activities					
24	You get so busy that you forgot where your child is and what he/she is doing					
25	Your child is not punished when he/she has done something wrong					
26	You attend parent-teacher association* (PTA) *meetings, parent-teacher conferences, or other meetings at your child’s school					
27	You tell your child that you like it when he/she helps out around the house					
28	You don’t check that your child comes home at the time she/he was supposed to					
29	You don’t tell your child where you are going					
30	Your child comes home from school more than an hour past the time you expect him/her					
31	The punishment you give your child depends on your mood					
32	Your child is at home without supervision					
33	You spank your child with your hand when he/she has done something wrong					
34	You ignore your child when he/she is misbehaving					
35	You slap your child when he/she has done something wrong					
36	You take away privileges or money from your child as a punishment					
37	You send your child to his/her room as a punishment					
38	You hit your child with a belt, switch, or other objects when he/she has done something wrong					
39	You yell or scream at your child when he/she has done something wrong					
40	You calmly explain to your child why his/her behavior was wrong when he/she misbehaves					
41	You use time out (make him/her sit or stand in a corner) as a punishment					
42	You give your child extra chores as a punishment					
